# Social Non-profit Bioentrepreneurship: Current Status and Future Impact on Global Health

**DOI:** 10.3389/fpubh.2021.541191

**Published:** 2021-10-01

**Authors:** Amir H. Sadeghi, Charlotte Koldeweij, Grissel Trujillo-de Santiago, Milad Tannazi, Nikkie Hosseinnia, Oscar van Loosbroek, Amir Manbachi, Yannick J. H. J. Taverne, Ad J. J. C. Bogers, Mario Moisés Alvarez

**Affiliations:** ^1^Biomaterials Innovation Research Center, Department of Medicine, Brigham and Women's Hospital, Harvard Medical School, Cambridge, MA, United States; ^2^Harvard-MIT Division of Health Sciences and Technology, Massachusetts Institute of Technology, Cambridge, MA, United States; ^3^Department of Cardiothoracic Surgery, Erasmus University Medical Center, Rotterdam, Netherlands; ^4^Avicenna Foundation, Avicenna Excellence Program, Utrecht, Netherlands; ^5^Department of Medicine, Utrecht University, Utrecht, Netherlands; ^6^The Helix Centre for Design in Healthcare, Institute of Global Health Innovation, Imperial College, London, United Kingdom; ^7^Microsystems Technologies Laboratories, Massachusetts Institute of Technology, Cambridge, MA, United States; ^8^Centro de Biotechnologia-FemSA, Tecnologico de Monterrey, Monterrey, Mexico; ^9^Department of Pharmaceutical Science, Utrecht University, Utrecht, Netherlands; ^10^Center for Bioengineering Innovation and Design, Department of Biomedical Engineering, Johns Hopkins University, Baltimore, MD, United States; ^11^Department of Neurosurgery, School of Medicine, Johns Hopkins University, Baltimore, MD, United States

**Keywords:** entrepreneurship, biotechnology, social entrepreneurship, non-profit, global health, bioentrepreneurship, affordability

## Abstract

For-profit biotechnological and pharmaceutical companies have played an essential role in the research and development (R&D) of innovative medical products and drugs for many decades and embody a trillion-dollar industry. The past decades have been marked by an increase in growth of social non-profit biotechnology companies and organizations led by entrepreneurs committed to solve (global) health issues. In this review, we define the concept of social bioentrepreneurship and consider the potential impact of such ventures on global health. We analyse the current status of non-profit biotechnology and clarify the strategy, motivation, funding, and marketing techniques of these enterprises. We find that these non-profit ventures mainly focus on neglected and rare diseases by using different but also similar funding, marketing, and business strategy approaches to for-profit biotechnology enterprises. We also identify good leadership, multidisciplinary teams, and public awareness as key components to achieve long-term survival and higher success rates. Challenges faced by bioentrepreneurs include the lack of a clearly defined regulatory environment or governmental incentives to support their endeavors. Overall, with this qualitative data review and market analysis we draw a promising picture of social non-profit bioentrepreneurship and underscore its current and future impact on global health issues.

## Introduction

For-profit biotechnological and pharmaceutical companies have played an essential role in the research and development (R&D) of innovative medical products for many decades and embody a huge industry. Total global spending on medicines alone was forecasted in 2013 to grow to $1.2 trillion in 2017 (1) Biologicals account for ~20% of this industry and the development of biological products is, despite the higher failure rate and uncertainty, growing faster than its counterpart, the small molecules (1, 2).

The past two decades have been marked by a significant growth in the creation of non-profit organizations. From 1993 to 2010, the proportion of scientists and engineers working in non-profit organizations in the U.S. nearly doubled from 5.8 to 10.7% (3). This trend also applies to the biotechnology sector. Non-profit biotechnology companies and their founders tend to fill a gap in the global health sector where there is a high potential social impact to be gained but little room for profit. By combining social entrepreneurship and biotechnology, this phenomenon can simply be coined “social bioentrepreneurship.”

Social bioentrepreneurship can be defined as an enterprise-model strategy that devotes efforts to solve health problems derived from social issues. A “Social Bio Start-up” may be interested in providing solutions for major global health issues such as HIV/AIDS (4), neglected tropical diseases, rare diseases, and non-affordable treatments, among others. Such conditions are rarely addressed by for-profit organizations. The financial model of a social bio-start-up can be non-profit, for-profit, or a more complex scheme that involves a combination of these models. Importantly, however, social bio-enterprises have to clearly indicate that they are a need-driven undertaking, as opposed to profit-driven.

The purpose of this paper is to further define the concept of mainly non-profit social bioentrepreneurship and explain its potential future impact on global health. We will investigate both bioentrepreneurs themselves and their non-profit biotechnological ventures. We will highlight the differences between for-profit and non-profit biotechnology, exploring the motivation of social bioentrepreneurs and examine the focus, strategy, funding, and marketing of non-profit biotechnology companies. Our focus will mainly be on non-profit companies that develop and commercialize diagnostic, preventive, and therapeutic medical products and drugs. This will be complemented with a qualitative market analysis and examples, and eventually, we will provide and discuss a future perspective on the impact of these non-profit enterprises on global health.

## Motivated by Impact: the Social Bioentrepreneur

Although science and technology have progressed exponentially over the last decades, the knowledge and tools available to most people living in developed countries have impacted the life of a minority of residents of less well-endowed nations. Millions of people around the world die due to diseases that would not be lethal if they had access to proper prevention, diagnosis, and/or treatment. Respiratory infections, heart disease, diarrheal diseases and diabetes mellitus are clear examples of this problem; they are some of the top 10 leading causes of death in the world (4) and are considered top priorities to be addressed by International Foundations to improve global health (5). While low-income countries are the most affected by these diseases, residents of middle- and high-income countries are far from being exempt from these health issues (see Table 1) (4). Historically, philanthropists have played an important role in addressing health shortfalls derived from social issues, however, their impact is limited to the extent of resources. An enterprise model might constitute a more sustainable way to bring long-term and effective solutions to these problems in the form of products and services. According to this view, a “Social Bioentrepreneur” may fit this purpose.

**Table 1:** The top 10 health challenges in high, upper-middle, and lower-middle income countries, and the priority health challenges addressed by the Gates Foundation.

**Health challenge**	**High income countries**	**Upper-middle income countries**	**Lower-middle income countries**	**Low income countries**	**Priorities Gates Foundation**
Lower respiratory infections	6	6	3	1	✓
HIV/AIDS			7	2	✓
Diarrheal diseases			5	3	✓
Stroke	2	1	2	4	
Ischemic heart disease	1	2	1	5	
Malaria				6	✓
Preterm birth complications			6	7	✓
TB			9	8	✓
Birth asphyxia and birth trauma				9	✓
Protein energy malnutrition				10	✓
COPD (^*^)	5	3	4		
Diabetes	8	5	8		
Cirrhosis of the liver			10		
Trachea-bronchus and lung cancers	3	4			
Road injuries		7			
Hypertensive hearth disease Orphan diseases	9	8			
Liver cancer		9			
Stomach cancer		10			
Alzheimer and other dementias	4				
Colon-rectum cancers	7				
Breast cancer	10				

*Numbers indicate the position in the top 10 ranking*.

* *Chronic obstructive pulmonary disease*.

### The Motivation of Social Bioentrepreneurs

“*We are a new generation of entrepreneurs, driven to solve some of the problems of society. So, we don't look at money or profit as the motive*.” These are the words of Krishna M Ella (6), a US-trained scientist that went back to India and started his journey as an entrepreneur by developing a vaccine for Hepatitis-B at a very low cost.

**Figure 1:**
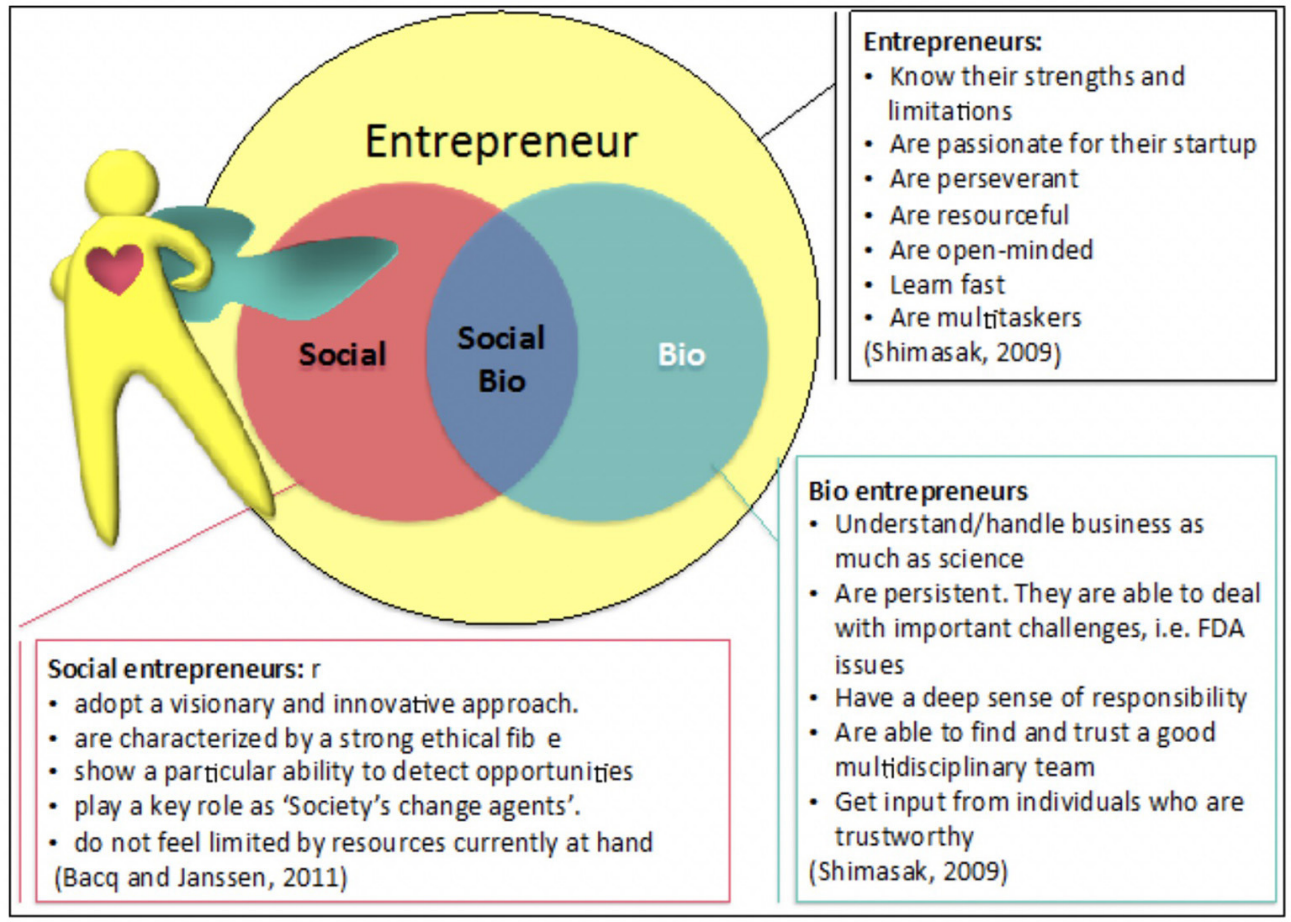
The social bioentrepreneur profile results from the merging of three sets of characteristics corresponding to an entrepreneur, a bio entrepreneur and a social entrepreneur. The social bioentrepreneur needs a solid entrepreneur base, strong skills of a bioentrepreneur, and be driven by the social- entrepreneur motivation. Here the social bioentrepreneur is personified as a single individual, but the skills or characteristics may be covered by a several people in the same team/enterprise.

As previously stated, the motivation to solve important health issues over that of gaining large profits appears to be the main characteristic distinguishing social bioentrepreneurs from other kinds of entrepreneurs. Social bioentrepreneurs however, need a wider set of characteristics to be successful (7). Figure 1 shows some of the key features of successful entrepreneurs, social entrepreneurs, and bioentrepreneurs. Social bioentrepreneurs can be seen as a merger between a social entrepreneur and the bioentrepreneur, inside the circle of entrepreneur, meaning they need the combined set of characteristics of the three types of entrepreneurs in order to provide non-profit solutions for global health issues. Oversimplifying, a Social Bioentrepreneur needs the ambition and cleverness of an Entrepreneur (8), the sensitivity of a Social Entrepreneur (i.e., altruism, integrity, trust in others, and empathy) (9, 10) and the scientific and academic knowledge of a Bioentrepreneur (11, 12).

### The Background of Social Bioentrepreneurs

Metha (13) named several examples of individuals with different backgrounds that funded successful biotechnology-based startups. Examples include (1) a large pharmaceutical executive with no scientific experience who teamed up with a scientist, (2) a sales executive trained as a physician, (3) a management consultant who partnered with a scientist, (4) a biology bachelor graduate, (5) an investment banker, (6) a scientifically minded entrepreneur and his wife, and (7) a physician who teamed with an entrepreneur. While in his paper Metha (13) noted that most biotechnology startups were founded by life scientists; the chances of creating success appear to increase when founded by a multidisciplinary team comprised of individuals with different backgrounds (13).

There is no stereotype that defines a potential social bioentrepreneur. Perhaps the most common denominator among them is a strong will to solve a health/social issue through an enterprise-model, using technical/scientific resources (from him/herself or a team) and addressing such problems in an effective and innovative way (Figure 1).

### Social Biotechnology Companies, Their Niche Markets, and Partners

We have studied the geographic distribution and founding years of social bioentrepreneurship ventures and non-profit biotech companies. In the analysis of 10 of these companies, we found that their headquarters were mostly (90%) located in the US. Only one company was located in Europe and had been founded in Switzerland (Drugs for Neglected Diseases). One of the US-based firms also had offices located in South Africa and China (AERAS). A possible explanation for the US-centricity of bioentrepreneurship ventures might be the high concentration of biotechnology companies and (academic) research institutes in the US, also a central location for R&D of biotech products. Persidis stated that the “*United states is at the forefront as the most desired destination for bioentrepreneurship”* based on an analysis of the generation of biotechnology products (14). Nevertheless he also stated that “*Imagination and drive, and not just location, will likely move biotechnology toward geographic parity.”* Imagination and drive are also key factors in the motivation of social bioentrepreneurs who are driven to solve global-healthcare problems. In the current context of global connectivity and with a clear trend toward the democratization of scientific tools, it can be expected that the distribution of social bioentrepreneurs will also move toward greater geographic parity.

**Figure 2:**
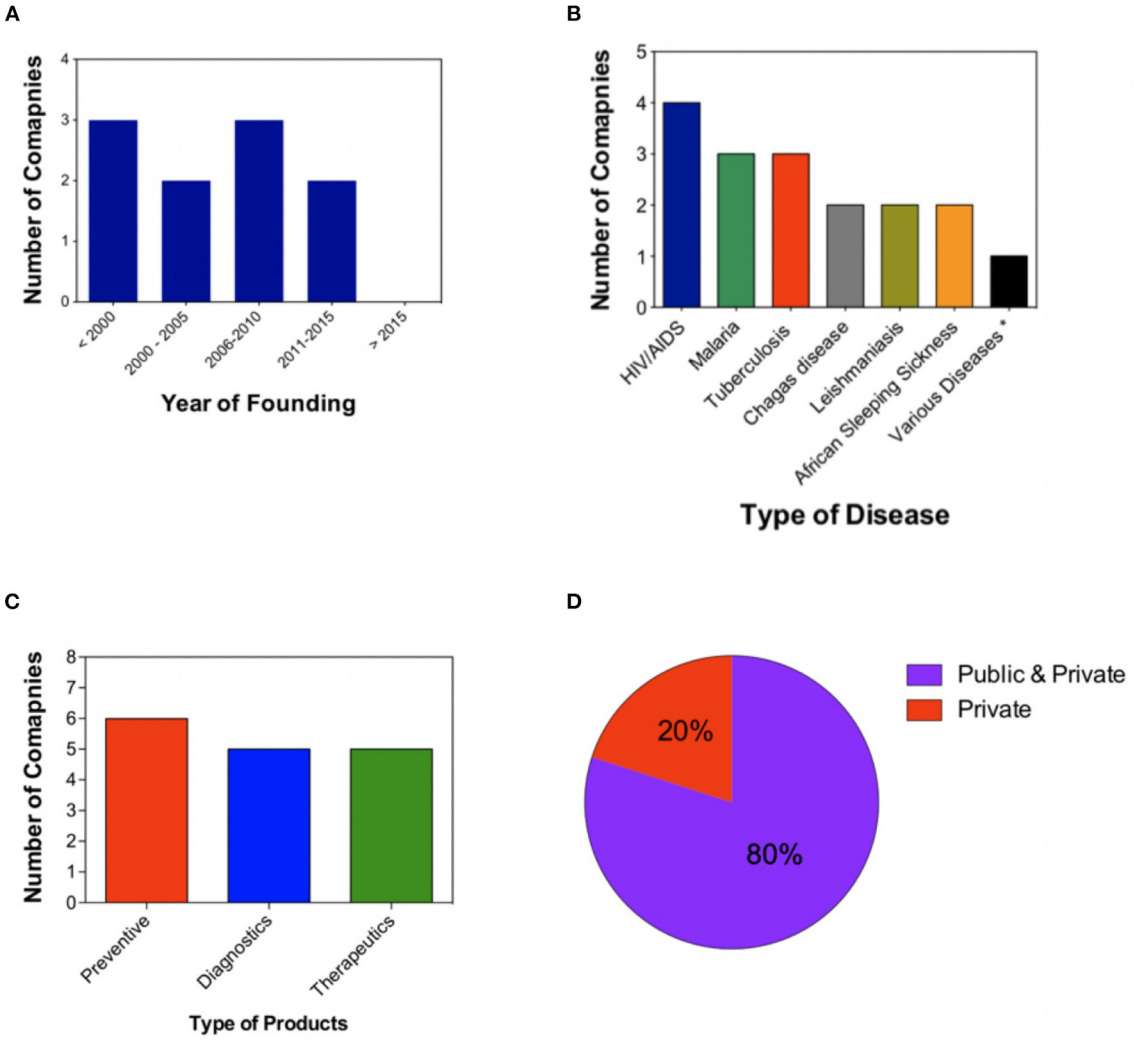
A brief analysis of social biotechnology companies. **(A)** Year of founding of ten different social biotechnology companies. **(B)** Diseases of focus. Quantitative representation of the diseases of focus of non-profit biotechnology companies. **(C)** Distribution of the types of products (preventives, diagnostics, and therapeutics) developed by social biotechnology companies. **(D)** Quantitative representation of the analysis of partners. ^*^Amebiasis, diabetes, child malnutrition, toxoplasmosis, onchocerciasis (river blindness), loiasis, human African trypanosomiasis (HAT), lymphatic filariasis, tetanus, measles, hypertension, and filarial disease.

In order to identify potential trends in the establishment of non-profit biotech companies, we analyzed the year of founding of such companies. Our analysis shows that within the last 18 years (2000–2018), seven of these ventures were founded and only three out of 10 originated in the years 1956 (Pacific Northwest Diabetes Research Institute), 1976 (Center for Infectious Disease Research), and 1974 (JHPIEGO; Johns Hopkins Program for International Education in Gynecology and Obstetrics) (Figure 2A). The last two decades have thus been marked by a significant increase in the foundation of non-profit biotechnology companies.

As stated earlier, the target niche markets of social biotechnology companies cover a broad range of medical conditions including neglected diseases (e.g., lymphatic filariasis, leishmaniasis, and Chagas disease), diseases such as HIV/AIDS, tuberculosis, and malaria, all in the top 10 causes of death in low-income countries according to the world health organization (Table 1 and Figure 2B) (4). To help address these problems, non-profit companies have been investing in the development of several type of products, ranging from diagnostics (e.g., diagnostic devices for HIV and hepatitis C, by Diagnostics for all, USA) to preventives (e.g., tuberculosis vaccines by AERAS, USA), and therapeutics (e.g., anti-malaria drugs, by Drugs for neglected diseases, Switzerland). A quantitative analysis of the companies that have been investing in these kinds of products is displayed in Figure 2C. To achieve their non-profit goals, non-profit biotechnology companies team up with a variety of partners such as industrial companies, universities, governmental research institutes, and foundations (e.g., the Bill and Melinda Gates foundation). As shown in Figure 2D, most of the companies we analyzed have partnered with both public and private institutions.

## Designing a Successful Strategy for a Social Biotechnology Company

For-profit biotechnological and pharmaceutical companies tend to focus their R&D efforts on the improvement of (chronic) medical conditions that mostly affect large populations in the developed world. On the other hand, non-profit biotech companies are increasingly being established to contribute to socially relevant medical innovations associated with low/non-profit markets. The business models of for-profit biotech firms are mainly determined by profit, whereas models of non-profit companies are based on a collaborative commitment to contributing to global health and social good. In general, non-profit biotech companies are focused on the development of medical products and services for diseases that affect the developing world and smaller patient populations. But how are these firms organizationally structured and what are critical drivers for a successful non-profit biotech company?

Determining features in the model adopted by a non-profit biotech company include its organizational structure, choice of portfolio, and fundraising strategy. We discuss important similarities in the structural and operational characteristics of non-profit bioentreneurship ventures and discuss how they may differ from for-profit enterprises.

In a climate of declining productivity (15), pharmaceutical R&D spending has kept soaring over the past few years (16). To ensure full recovery from high R&D investments and other associated costs, the development and commercialization of a new pharmaceutical product is typically backed by significant investments in intellectual property (IP) protection (17). Relying on a much smaller available budget, non-profit biotech companies can lessen their R&D costs by using alternative methods for safeguarding their IP. For example, patented drugs can be donated or licensed out by both academia and pharmaceutical companies (18). The advantage of a donation is that the non-profit company can use these patents to develop new drugs, while the donor company benefits in terms of tax deduction (19). In addition, repurposing or reusing off-patent drugs that have been approved as a treatment for other diseases is an alternative for bypassing early stage drug development (19, 20). By searching for effects that are off-target, an existing drug compound can be repositioned to treat other diseases. As a result, these drugs can immediately be tested in a phase II and III clinical trial and thereby reduce the overall R&D costs by ~40% (19, 21).

Although the motives and financial interests of non-profit companies differ from traditional pharmaceutical companies, they also have similarities when it comes to their structural organization. Like large pharmaceutical companies, a non-profit biotech company requires a scientific board with broad-ranging expertise in topics such as (pre-) clinical pharmacology, toxicology, and regulatory affairs. In addition, a non-profit biotech company should have a leadership team that is capable of designing and interpreting the results of clinical trials. Partnerships with academia, for-profit companies and the government could help stimulate collaborative initiatives in the R&D of neglected diseases (22). This collaborative approach between public organizations and private firms, termed public-private partnerships (PPPs), has been found to be valuable in the development of new drugs. Examples of successful PPPs in drug R&D include the Medicines for Malaria Venture (MMV), the Global Alliance for Tuberculosis Drug Development (GATB), the Drugs for Neglected Diseases Initiative (DNDi), and the Institute for One World Health (iOWH) (22). Such collaborations could also aid the manufacturing and distribution of new medical products in other low resource countries (19).

Establishing non-profit biotech companies involves obtaining a non-profit status as a 501 *(c)(3)* charitable organization in the US (23). Capitalizing on this tax-code structure allows these companies to choose their focus (portfolio) of R&D based on global need instead of the financial interests of their investors. Flexibility in portfolio development plays an important role in the design of a successful strategy of a non-profit company, as illustrated by several companies (Table 2). By focusing on a diverse set of populations, neglected and orphan diseases, a wide variety of compounds and drug targets can be developed to maximize social value rather than financial profit. Non-profit companies' lower advertising and marketing expenses are another factor differentiating them from for-profit ventures (18). Such savings on marketing expenses come with a significant advantage in that the budget of social bioentrepreneurship companies can be used more effectively toward the development of new products. Moreover, non-profit organizations and biotech companies are often dependent on scarce and limited funds from philanthropic organizations and institutes such as the Bill Gates Foundation (5). Consequently, a good understanding of the funding opportunities and funding models are necessary for setting-up and running a successful non-profit biotech organization, which will be discussed in the next section.

**Table 2:** Funding sources of several social bioentrepreneurship organizations.

**Organization, place (year founded)**	**Place and year of foundation**	**Mission**	**Revenue sources**	**Funding in 2015**	**References**
Drugs for neglected diseases (DND*i*)	Geneva, Switzerland, 2003	DNDi focuses on developing new treatments for the most neglected patients suffering from diseases such as sleeping sickness, leishmaniasis, Chagas disease, malaria, filarial diseases, and pediatric HIV.	Relies on a combination of public (51%) and private (49%) funding.	Public funding: USD 25,335,240 Private funding: USD 13,859,833 Total: USD 43,283,345	(24, 25)
AERAS	Rockville, Maryland, USA, 2003	Pursuing affordable and globally effective vaccines for tuberculosis.	Funded by The Bill & Melinda Gates Foundation and multiple other public and private funds and grants.	Bill & Melinda Gates Foundation: USD 28,765,000 Other grants: USD 8,130,632 Contract service: USD 616,980 Total: USD 37,512,612	(26–33)
Diagnostics for all	Cambridge, Massachusetts, USA, 2007	A non-profit enterprise aiming to create low-cost, easy-to-use, point-of-care diagnostic devices designed specifically for the developing world.	Relies on a multiplicity of sources: universities (MIT), foundations (The Bill & Melinda Gates Foundation), governmental (USAID, DARPA, the Government of Norway, Grands Challenges Canada, DFID, Koica) as well as private (Merck).	Saving Lives at Birth Seed (a partnership between USAID, DFID, the World Bank, the Bill & Melinda Gates Foundation, the Government of Norway and Grands Challenges Canada), grant: USD 250.000 Total: unknown	(34, 35)
Institute for Pediatric Innovation (IPI)	Boston, Massachusetts, USA, 2006	Develops safe pharmaceutical formulations and medical technology for children.	Funded by hospital membership (42%) royalties of product (30%) and other public and private sources (28%).	Hospital consortium membership: 262,500, royalties of Epaned product 188,300, Grants, donation, sponsors and others: 192,000. Total: $640,000	(36–38)

## The Right Resources for Non-Profit Bioentrepreneurship Enterprises: Raising Capital and Training the Next Generation of Healthcare Entrepreneurs

Non-profit bioentrepreneurship ventures, like any other form of enterprise, are reliant on at least three types of capital in order to establish themselves as a successful undertaking, namely financial capital, human capital and social capital. In this section, we examine all three forms of capital, social capital being also addressed in the next chapter on public awareness of non-profit biotech ventures. The following questions are posed: (1) what are important sources and models of funding for social bioentrepreneurs? (2) in what ways do academic institutions contribute to the rise of social bioentrepreneurship through their curricular offer? (3) what other types of non-financial support can non-profit bioentrepreneurs rely on?

### Funding Sources for Social Bioentrepreneurs

Faced with the challenge of building a sustainable non-profit enterprise, non-profit biotechnology entrepreneurs tend to leverage multiple channels of funding, relying on a mix of donations from individuals, foundations and philanthropists (39), public governmental and non-governmental grants, as well as corporate funding and venture capital, among other sources of finances (40). This is illustrated in Table 2, detailing the funding sources of a number of non-profit biotechnology enterprises.

As illustrated in Table 2, non-profit biotechnology ventures often remain primarily dependent on governmental and foundation money for their finances. Key public funders include the United States Agency for International Development (USAID) and the UK Department for International Development (DFID), and the Bill and Melinda Gates Foundation on the non-governmental side. This dependence on public and foundation money appears typical of the non-profit sector, characterized by high investment risks and hard to measure outcomes (41). The impact of a non-profit biotechnology venture, furthermore, is primarily derived from the social value it creates a dimension of success that remains hard to thoroughly capture through quantitative approaches (42–44).

Despite their reliance on public funding, non-profit bioentrepreneurs are also leveraging private investment as a means to diversify their sources of financial capital. Alongside more traditional forms of private funding and non-financial support as part of corporate social responsibility (45), biotech entrepreneurs can also leverage corporations and the venture capital market's growing interest in novel forms of engagement with societal enterprises, ranging from impact investment to venture philanthropy (46, 47).

Increasingly, non-profit bioentrepreneurs have also started including less traditional sources of finances in their funding mix. Novel funding strategies leveraging individuals', including high net worth individuals' (47), morals through digital crowdfunding campaigns to gain private donations (48), as further discussed in the next chapter and making use of new tailored forms of investment for higher risk enterprises, examples including startup incubator money or social venture capital (44, 49, 50). While the growing reliance of bioentrepreneurs on non-traditional forms of finance tend to blur the boundaries between for-profit and non-profit ventures (51, 52), the latter sources of funding still constitute a limited share of the total mix of financial revenues for social biotech entrepreneurs. Potential reasons for this including the lack of structure and temporary scope of crowdfunding initiatives, unfit to fund non-profit initiatives over the long run, and the greater selectivity and focus of incubators and social venture capitalists on for-profit initiatives with benefits for society, favoring a *double bottom line* return (41).

The hybrid, but still largely public and charity-based mix of funding characterizing non-profit biotechnology ventures can be seen in the light of the various sizes and objectives of such organizations, also illustrated in Table 2. From running local community health clinics in high income countries to addressing global health issues like neglected tropical diseases or organizing prevention campaigns for sexually transmitted diseases and domestic abuse, the funding requirements and most appropriate financing instruments are likely going to be varied, especially when considering the survival of such ventures over time.

### Models of Funding for Non-profit Healthcare Initiatives: No One-Size Fits All

Given the heterogeneity characterizing the financial landscape of social bioentrepreneurship ventures, one may wonder whether some distinctive models of funding can be identified. A 2009 article by Foster *et al*. in the *Stanford Social Innovation Review*, listing 10 funding models commonly used by 144 non-profit organizations created since 1970 and having grown by over USD 50 million a year, offers an interesting theoretical framework in this view (53). We discuss some of these funding models that depict the strategies most commonly employed by non-profit enterprises in the healthcare sector. First among the funding strategies discussed here, the *Heartfelt Connector* approach allows non-profit organizations to garner revenues by focusing on causes that resonate with the concerns of a large number of individuals. The Susan G. Komen Foundation is an example of an organization using this approach as a means to fund research, healthcare, and advocacy efforts against breast cancer (54). The *Big Bettor* approach is another tactic whereby an organization, often involved in medical research, like the Stanley Medical Research Institute, seeks to gain the support of a major donor, either a foundation or a well-endowed individual. The *Beneficiary Builder* constitutes a third approach leveraged by non-profit ventures like the Cleveland Clinic that call for donations from specific individuals who benefitted from their services in the past. The *Resource Recycler* approach employed by the likes of the AmeriCares Foundation illustrates another type of strategy whereby a non-profit venture operates by redistributing in-kind donations from individuals or industrial corporations to its targeted beneficiaries. *Policy Innovators* like FHI 360, with expertise in family planning and reproductive health, make compelling appeals for government funding by crafting successful approaches to address critical public health issues. *Market Makers* like the American Kidney Fund, lastly, seek to glean donations as a means to fill a market gap that cannot ethically be addressed through a for-profit strategy, access to dialysis for low-income patients constituting a prime example of this.

While providing useful archetypes to support a better understanding of the funding approaches commonly adopted by social bioentrepreneurship ventures, Foster et al.'s funding models are neither comprehensive nor fully representative of the variety of funding sources often combined within a single non-profit healthcare organization, as also applies to the various institutions cited as examples in the Stanford Review. Ultimately, there cannot be a single recipe for success when it comes to funding non-profit biomedical initiatives of such a wide variety of nature and scope.

### The Status of Educational Programs on Social Bioentrepreneurship

Reflective of the importance of funding and fundraising strategies for non-profit leaders, the latter subjects have become core components in the curriculum offered by academic institutions seeking to help train the next generations of social bioentrepreneurs. An example of the growing variety of courses on bioentrepreneurship (55), the Stanford Biodesign program has started offering a course on “Global Biodesign: Medical Technology in an International Context” which encourages students to work in teams with real-world companies in order to develop business plans for introducing existing products from one country into a new global market (56).

The Johns Hopkins Center for Bioengineering Innovation and Design (CBID) program offers a master's program requiring students to travel to countries such as India, China, Uganda or Rwanda to identify a healthcare problem that can be addressed through innovation (57). A number of these teams are now successful viable entities working with international companies to develop their products. Following their graduation, some of the more advanced teams have started working with Jhpiego (58), an international non-profit health organization affiliated with Johns Hopkins University, to distribute their products in international settings.

Instituto Tecnológico de Monterrey, the largest private university in México, has launched various initiatives to promote the participation of students and faculty members in social ventures. Among such actions, students and mentors plan and execute actions to alleviate pressing health or medical issues in remote or unprivileged communities. While such programs are planned to last only a few weeks to months, the hope, through this training, is for students “to be able to use (their) entrepreneurial spirit to generate innovative companies with high social impact” following their graduation (59).

Other examples of graduate courses and university-based incubators focusing on entrepreneurship in health and the life sciences can be found in institutions like the Karolinska Institute in Sweden (60). Amsterdam Free University in the Netherlands, the University of New South Wales in Australia (61), or the University of Cape Town in South Africa (62). While funding for such courses remains limited, the growing emphasis on integrating multidisciplinary bioentrepreneurship programs in the curriculum of reputed academic institutions across a large number of countries will likely help train new generations of entrepreneurs with the ability to develop innovative solutions and to deploy new markets for medical products and services by relying on ingenious fundraising mechanisms (55).

### Non-financial Support to Social Bioentrepreneurship: A Nascent Infrastructure

Alongside their reliance on financial and human capital in the form of public and private funding and dedicated educational programs, the long-term success of social bioentrepreneurship ventures' is in large part conditioned by the existence of a favorable institutional environment. A resource-constrained entrepreneur's ability to innovate and to compete with more profitable enterprises will depend on the existence of the right kind of policy support, regulatory and fiscal incentives, as well as best-in-class infrastructure for research & development, market access, quality insurance, and scale-up.

Despite the growing awareness of certain governments, especially of developing nations like India (63), in this view, the failure of the latter governments to create a legislative, regulatory and fiscal environment fostering the growth of non-profit actors in the biotechnology space remains patent (63–65). Despite their theoretical, and in some cases, financial backing of social biotechnology endeavors in view of their relative affordability, civil servants and public institutions in numerous countries remain at a loss when it comes to ensuring the provision of holistic support to help such ventures establish themselves at scale. The same is true when it comes to incentivizing other actors, namely in the private and non-governmental sectors, to make their resources and expertise available to social bioentrepreneurs that could build on them (64, 66).

Recent but still relatively rare initiatives in that sense include that of the European Medicines Agency which currently provides free scientific advice to medicine developers through its Committee for Medicinal Products for Human Use (67). The Medical Research Commercialization Fund in Australia and New Zealand, a collaborative platform gathering various organizations including the Australian and New Zealand governments, several Australian state governments and public Australian pension funds, and a large number of academic hospitals and medical research institutes, is another organization seeking to facilitate market access for social healthcare entrepreneurs, among other actors in the biotech space. Through the Australian government's Accelerating Commercialization scheme (68), the latter actors can gain access to expert networks and receive up to half of eligible project costs to help them develop their ideas into marketable products (69).

## Reaching Out to the Public and Raising Awareness in Biotechnology

Reaching out to different audiences (e.g., public, private) using various communication means is key for social entrepreneurs (70). Arguably, a good public image is even more critical for social bio-enterprises, which typically require more capital and operational resources to sustain healthy operations than other social ventures. Compared to other social entrepreneurs, bioentrepreneurs face the additional challenge of explaining to society what they do and why they do it while also being expected to properly and responsibly providing in biotechnology for the community well-being. The notion of social entrepreneurship and the commitment to solve a social problem is easy to understand and appealing for the general public (71–74). The positive connotation of a non-profit venture adds attractiveness to the proposition of a social enterprise. The inclusion of the *bio* prefix” on the contrary, may negatively influence the public perception of non-profit bioentrepreneurs. Perceptions of the word *biotechnology* vary widely among cultures, age groups, and depending on people's educational background and gender (75). Overall, a better knowledge of biotechnology correlates with a more favorable attitude toward biotechnology (75, 76). In addition, public perception strongly depends on the particular area of biotechnology being discussed. Health-related forms of biotechnology are significantly better received than, for example genetically modified organisms or food (GMOs/GMF) (77). Even in the health sector, those biotech social enterprises dedicated to biopharma/pharma ventures could possibly be affected by negative opinions of the pharmaceutical industry, for example in the U.S. where large pharmaceutical groups have recently been criticized for a range of behaviors perceived as pervasive misconduct.

**Figure 3:**
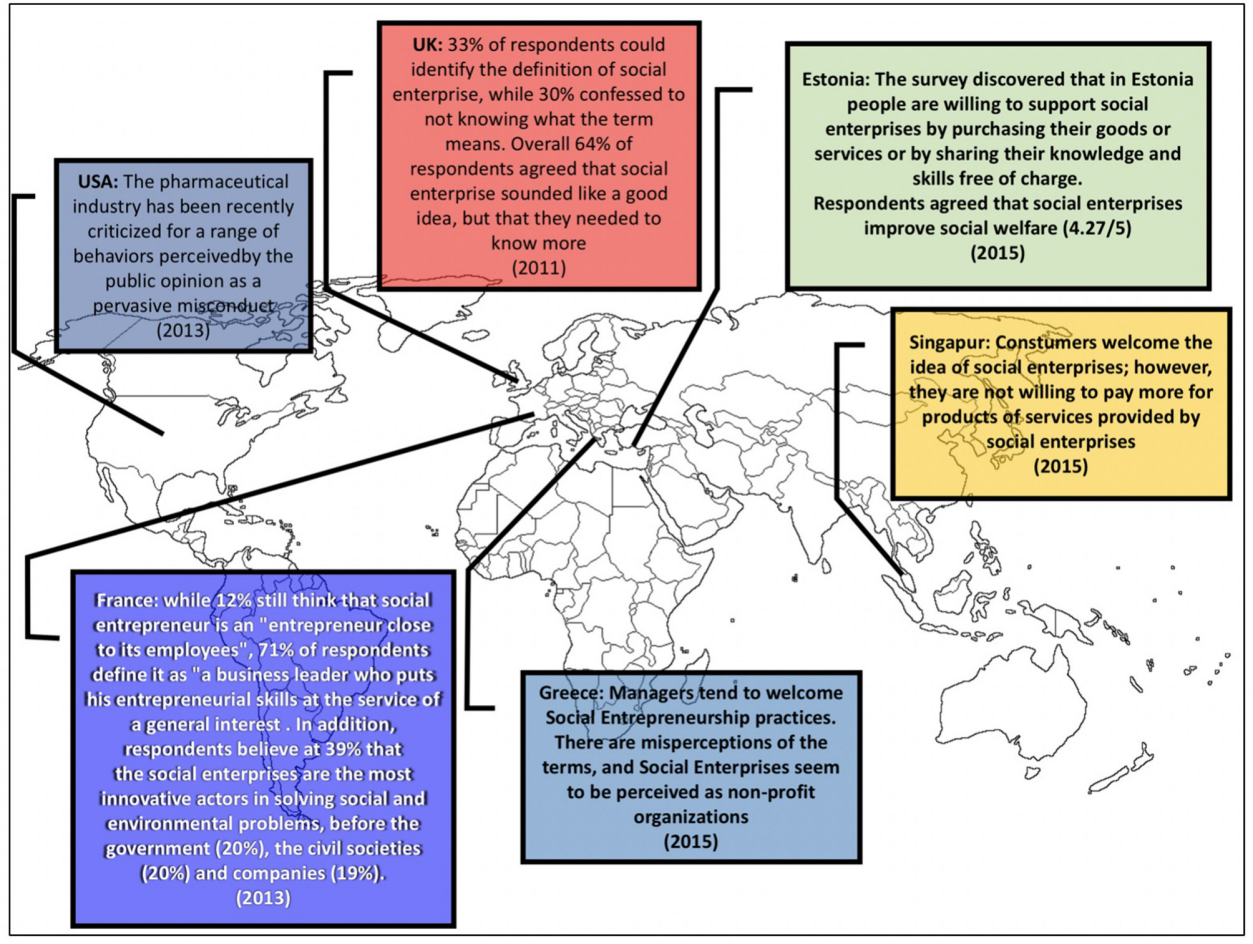
Perceptions related to “social enterprises” in different countries.

**Figure 4:**
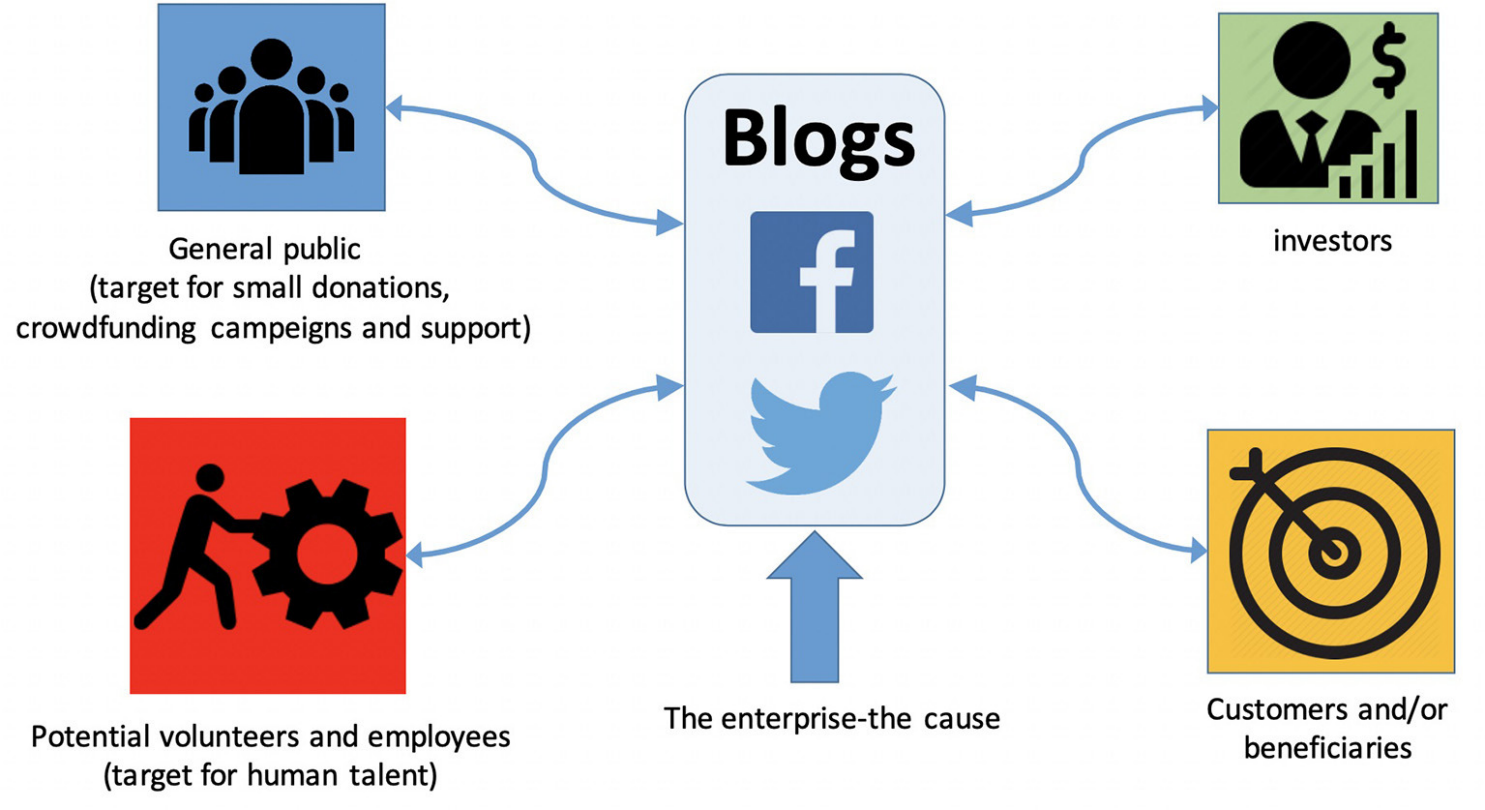
Mass media and target publics for a social bioentrepreneur. Blogs and social media are the virtual/international headquarters of social bioenterprises, they are useful to engage (more than only communicate), to emphasize the cause and to show the strength of the community network. They also serve as platforms for interaction, and documentation.

All in all, given the positive perception of social entrepreneurs and the fact that most social bioentrepreneurs target health items that are highly relevant for society, the public opinion of social biotechnological entrepreneurship is expected to be more positive than for any other biotechnology related concept (Figure 3: “map of perceptions”). Social bioentrepreneurs should capitalize on this positive perception (71). Conventional communication platforms such as television, the printed press and radio are important resources to position a social enterprise in the public opinion. In today's highly digitalized world, Twitter, Facebook and blogging among other social media resources are probably the most cost-effective strategy that non-profit social enterprises can rely on to reach a wide diversity of audiences (Figure 4). Additionally, blogs are a particularly useful communication resource for social entrepreneurs. A professional blog can serve as the virtual headquarters of a social enterprise, a reservoir of information and documentation, and a cost-effective means to reach investors, clients, and the general public alike. Blogs are easy to manage and use for the entrepreneur and his or her audience; they provide full and flexible control over the content; and they are a cost-efficient way to communicate a vision and engage the audience (78).

It is intuitive to assume that there is a definite link between successful enterprises and an established social media presence. Some basic strategies may be helpful to maximize the impact of a social bio initiative. People relate to a cause more easily than to an organization. Actively advertising the cause of a social bioenterprise is key to position it well in the public opinion. The problem to be solved should be the central element on any communication campaign in social media, not the organization nor the founder. Today, building an audience for a cause largely occurs online; to keep a close and interactive contact with the public is one of the largest challenges of social bioentrepreneurs. Engagement is crucial to sustain healthy on-line communities; and a high number of followers on Twitter and Facebook does not necessarily imply engagement on its own.

## Success Stories: Creation, Strategy, and Impact

Initiatives founded by social bioentrepreneurs can become successful organizations and make a significant contribution to improving global health. Two cases from our analysis will be outlined from founding to current activities to illustrate how the aforementioned theory can be put in practice and to explore which factors, whether internal or external to the venture, are integral to success. Both cases have distinct inception stories and serve different patient groups with unmet medical needs.

The Initiative for Pediatric Innovation (IPI) was founded in 2006 by Donald Lombardi, an expert in technology transfer, and successfully addresses the unmet medical needs of children as a patient group. Performing clinical trials on a pediatric population is complex and associated with high costs and ethical barriers. Furthermore, the financial incentives for developing pediatric specific indications are limited due to a smaller market size (79). Recently, regulatory institutions have tried to mitigate this problem by introducing legislation. IPI's strategy revolves around co-development and stimulating collaboration. IPI partnered with Silvergate Pharmaceuticals to successfully develop Epaned, a pediatric friendly formulation of enalapril, a routinely used antihypertensive drug. More recently, IPI has collaborated with multiple pharmaceutical companies, including Pfizer, in efforts to develop pediatric medical devices and has helped founding the new England pediatric device consortium (NEPDC). IPI's other activities include organizing collaborations, conferences and education services, which also constitute the initiative's main source of income. IPI reported a total revenue of 640 thousand USD in 2015. The revenue for IPI came from consortium membership fees, royalties, income from service products, sponsorships, and donations (80). IPI is an excellent example of a non-profit enterprise that has successfully met the need of an underserved group.

In contrast to the IPI, the Drugs for neglected Disease initiative (DnDi) was founded in 2003 by seven different large institutions. The founding partners range from governmental organizations, including the Malaysian ministry of health, the World Health Organization to non-governmental organizations including Medicines Sans Frontières. The vision of DnDi was to fill a void left by market failure of ventures centered on the development of drugs for neglected diseases and develop easy to use, safe, affordable and effective treatments for patients suffering from these diseases (81). Although neglected diseases affect more than a billion people worldwide, most of these people live in rural areas, and providing them with access to appropriate treatments comes with significant logistical and organizational challenges. Many pharmaceutical companies regard neglected diseases as markets with low expected returns and instead focus their attention on more profitable therapeutic areas.

DnDi currently has six products on the market with more in the pipeline, including 13 new chemical entities (82). DnDi reported an income of 43 million USD in 2015, with most of its funding originating from public and private partners (81). With clinical trial sites and laboratories all over the world, DnDi tries to engage countries that are heavily affected by neglected diseases in order to build their capacity to tackle the problem locally in the future. While DnDi is a large player in the social biotech niche, the relatively small investments it makes to develop products hint at a very efficient organization. At the center of DnDi's strategy are partnerships and collaborations, which range from public universities to large pharmaceutical companies to civil right groups. For an excellent article covering DnDi's drug discovery and development strategy as well as decision making process please refer to reference (81).

Whereas, DnDi took on the risky development and distribution process for molecular entities requiring large amounts of financial support, IPI reformulates existing medications and fosters collaboration to serve unmet medical needs. These two cases highlight the possibility of alternative approaches to impact global health based on the unmet medical needs of patient groups. They also illustrate the flexibility of this niche industry, to which both individuals and large institutions could contribute. Furthermore, they serve as illustrations of the potential efficiency gains to be achieved through social biotech ventures relying on alternative—and significantly more affordable- costing models. These cases could serve as inspiration for social entrepreneurs, although it is perhaps too early to put forward a best-practice theory.

## Discussion

To the best of our knowledge, this review constitutes one of the first academic endeavors to define the concept of social bioentrepreneurship and the only one to shed light on social bioentrepreneurs' growing contribution to global health. By combining a market analysis with qualitative data on non-profit health biotechnology companies, we intended to draw a comprehensive review that was both practically and theoretically grounded. In addition to discussing the motivations, funding sources and market strategies of non-profit biotech companies, we identified some of the key factors likely to affect the survival and success of such enterprises over the short and longer run, with multiple examples at hand. This analysis had to be conducted based on a limited number of sources, a majority of which focused on enterprises headquartered in the United States. While part of this bias can be attributed to our non-systematic literature search, it is likely to reflect actual geographical imbalances described by other authors referred to earlier on in this review. Other explanatory factors include a shortage of data from non-Western countries as well as the varying or absent legal definitions of non-profit biotech companies and scarce funding available outside the United States (71). The novelty of social bioentrepreneurship and the *ad hoc* emergence of such enterprises meant we had to qualify an evolving and still highly heterogeneous field of practice that was virtually absent from academic scholarship and that sometimes appeared difficult to isolate as a distinct institutional form. The comparison with business models used by for-profit biotech enterprises offered a useful theoretical backbone in this view.

**Figure 5:**
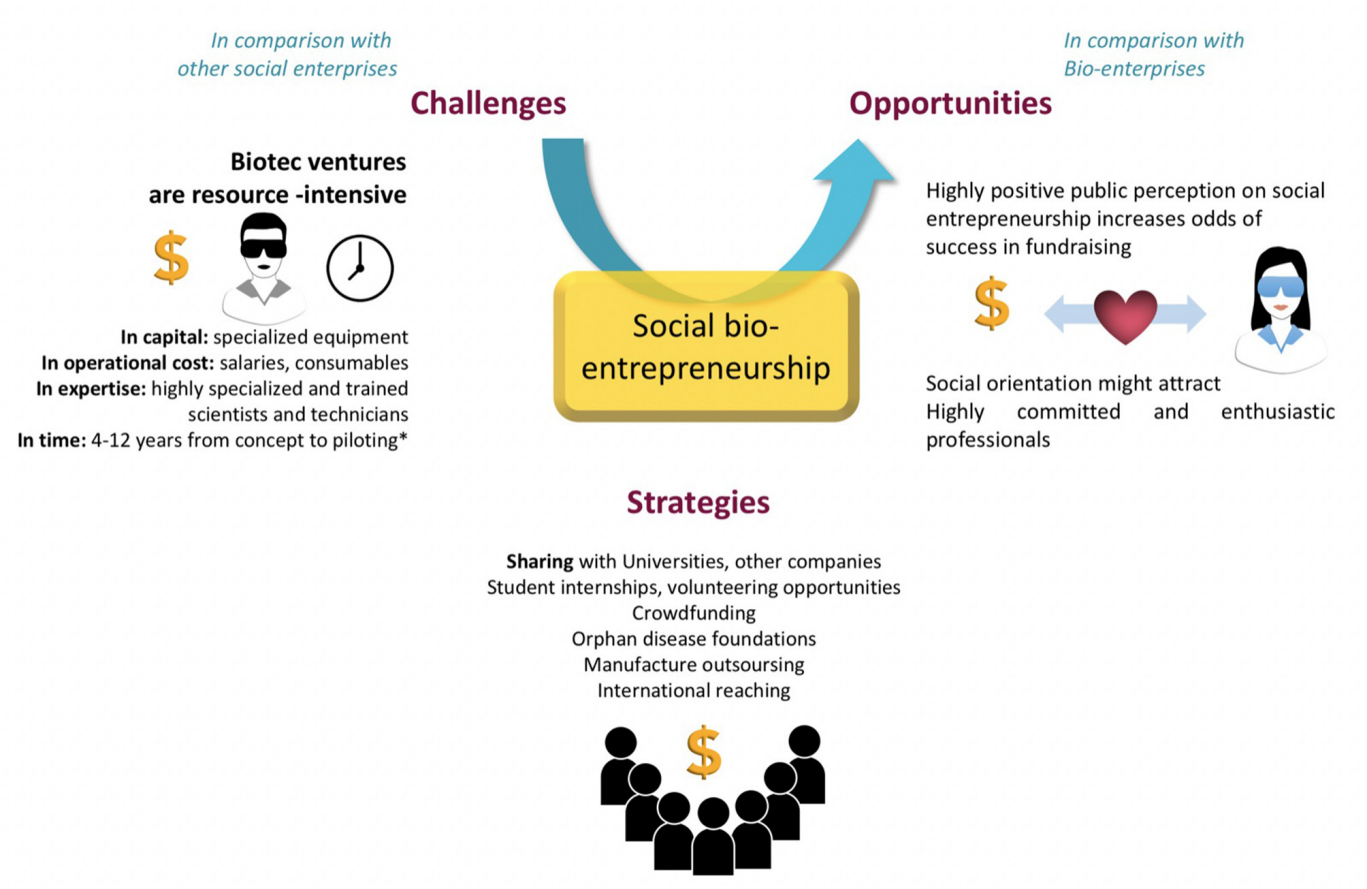
Challenges, strategies, and opportunities of social bioentrepreneurship as compared to social and bio-enterprises.

Having learnt more about the origins, motivations and the key factors of success of social bioentrepreneurs, it becomes possible to draw some reflections on their perspectives over the coming decades. While we expect the growth in non-profit biotech ventures to continue and to significantly impact global health in the near future, such ventures will have to creatively address a number of challenges (Figure 5) in the frame of a tightening context of global finance. Be it the effects of climate change, likely to be most hardly felt in the least developed economies and that may drive both governments and private investors to re-channel their funds toward urgent adaptation measures (83), or the global resurgence of nationalistic interests, prominent in and yet not limited to the United States (84), also a key source of funding for social bioentrepreneurs, the competition for financial resources is likely to keep growing over the coming years. Such challenges, however, might also be seized as a new realm of opportunities by non-profit biotech companies (Figure 5). In a context of dwindling funding, operating with a lighter and more flexible institutional form and offering more hands-on or locally tailored solutions than traditional NGOs might prove advantageous in allowing entrepreneurs to rapidly show a track record of success, likely to be valued by investors across the board. Climate change itself is expected to bring about large negative effects on humans' health and might form another potential niche market for social bioentrepreneurs provided that they choose to invest early and manage to successfully reach out to non-traditional funding sources like climate adaptation funds. Other health issues that remain currently shunned by for-profit companies due to low returns on investment like chronic ailments coming with population aging and soaring obesity rates may also prove appealing by affecting an ever-growing number of people across both developed and developing countries (85, 86). Addressing such market gaps is likely to come with profits that although low on a per capita basis, may prove sustainable as part of a non-profit economic model, even when focusing on non-traditional markets characterized by higher rates of market failure (87). Alongside more conventional sources of funding, social bioentrepreneurs have proven that they were well-positioned to leverage social capital at a time where the pharmaceutical industry and larger biotech start-ups were being chastised for their money-driven ethos and lack of transparency (88, 89), successfully appealing to individuals' support of a given cause through digitally backed funding schemes. All in all, having arisen in a challenging context in which they had to leverage both innovation and conviction to persist and thrive against highly profitable competitors, non-profit biotech companies might be at an advantage when it comes to devising creative business solutions that capitalize on their high social value while grasping the best opportunities at hand in the face of growing economic and regulatory uncertainty.

While this review underlines several factors of resilience for social bioentrepreneurs over the decades to come, some important questions and challenges remain for those interested in this type of venture, whether from a business or academic perspective. What are the opportunities and most pressing vulnerabilities of these enterprises in an increasingly competitive climate? How likely are non-profit biotech companies to invest in solutions addressing the neglected needs of developing world populations given the limited capital available to them, the lack of a clearly defined regulatory environment and incentives, and the financial risks and logistics involved? How many biotech ventures have been started by social bioentrepreneurs from technologically advanced and fast-growing economies like China or India and to what extent are the latter enterprises willing to depart from the business model and market strategies adopted by their Western competitors? Has digitalization reached a stage where such ventures could rely or train cheaper sources of human talent in countries likely to benefit the most from their efforts, for example in sub-Saharan Africa? Looking at social bioentrepreneurs' incentives and constraints, where are national regulations most favorable to non-profit biotech companies at this stage? To what extent are non-profit biotech companies seeking to influence the legal status quo on health-related innovation and intellectual property? What are some decisive factors for public and private investors to lend their money to these ventures, and in what direction are investors' potentially shifting preferences in the face of macro-economic changes likely to tip the competition, in terms of both products and geographies? Finally, to what extent will social biotech companies be able to monetize societal convictions and public sentiment as an additional source of funding?

## Author Contributions

This study was designed, directed, and coordinated by AS and MA. MA as the principal investigator provided additional conceptual and analytical guidance for all aspects of the project. AS, CK, GT, AM, NH, MT, and OL performed (market) analyses and contributed to the writing of separate parts of the manuscript under the guidance of MA and AS. AS, GT, MA, and NH have contributed to the design and preparation of the figures and tables. YT and AB in addition to all other authors commented and reviewed the manuscript. All authors have approved the submission of this manuscript.

## Conflict of Interest

The authors declare that the research was conducted in the absence of any commercial or financial relationships that could be construed as a potential conflict of interest.

## Publisher's Note

All claims expressed in this article are solely those of the authors and do not necessarily represent those of their affiliated organizations, or those of the publisher, the editors and the reviewers. Any product that may be evaluated in this article, or claim that may be made by its manufacturer, is not guaranteed or endorsed by the publisher.
